# DOCK2 protects against bacterial sepsis by constraining T helper 1 response

**DOI:** 10.3389/fimmu.2025.1527934

**Published:** 2025-05-29

**Authors:** Shusen Ye, Linzi Huang, Yuhao Zheng, Shanshan Liu, Xiangyang Wang, Haoyuan Yu, Lisi Zhu, Texi Liang, Yifei Wang, Chunmin Zhang, Fan Wu, Lilin Ye, Yingjiao Cao

**Affiliations:** ^1^ Guangdong Provincial Key Laboratory of Immune Regulation and Immunotherapy, School of Laboratory Medicine and Biotechnology, Southern Medical University, Guangzhou, China; ^2^ Institute of Immunological Innovation and Translation, Institute of Life Sciences, Chongqing Medical University, Chongqing, China; ^3^ Guangdong Cardiovascular Institute, Guangdong, Provincial People’s Hospital, Guangdong Academy of Medical Sciences, Guangzhou, China; ^4^ Department of Hepatic Surgery and Liver Transplantation Center, The Third Affiliated Hospital of Sun Yat-sen University, Guangzhou, China; ^5^ Guangdong Provincial Key Laboratory of Liver Disease Research, Guangzhou, China; ^6^ Pediatric Intensive Care Unit, Guangzhou Women and Children’s Medical Center, Guangzhou Medical University, Guangzhou, China; ^7^ Department of Neonatology, Guangzhou Key Laboratory of Neonatal Intestinal Diseases, the Third Affiliated Hospital of Guangzhou Medical University, Guangzhou, China; ^8^ Institute of Immunology, Third Military Medical University, Chongqing, China; ^9^ Department of Immunology and Microbiology, Zhongshan School of Medicine, Sun Yat-sen University, Guangzhou, China

**Keywords:** DOCK2, Th1, LPS, sepsis, *E.coli*

## Abstract

**Background:**

Sepsis is a systemic host response to infection with life-threatening consequence which ranks among the top ten causes of death worldwide. Nevertheless, our understanding of the molecular and cellular impact of sepsis remains rudimentary.

**Methods:**

A mouse sepsis model was established through LPS induction and *Escherichia coli* (*E. coli*) infection. Flow cytometry and enzyme-linked immunosorbent assay (ELISA) were used to detect T helper 1 (Th1) cell subsets and serum pro-inflammatory cytokines in septic mice. Additionally, in vivo neutralization experiments were conducted to block IFN-γ and CD4+ T cells, respectively, to explore the regulatory effect of DOCK2 on septic mice. Finally, the regulatory mechanism of DOCK2 was analyzed using an in vivo RNA-seq system.

**Results:**

We identified dedicator of cytokinesis 2 (DOCK2) is a critical downregulating factor for LPS signal pathways. DOCK2-deficient mice were highly sensitive to LPS-induced sepsis and *E. coli* sepsis with increased levels of inflammatory cytokines, especially IFN-γ which were mainly due to hyperresponsive Th1 cells. Ulteriorly, we verified the vital role of DOCK2-mediated Th1 cells in sepsis by neutralizing both IFN-γ and CD4 and found both of which blockade reduced the severity of sepsis in *Dock2^−/−^* mice. Mechanically, DOCK2-mediated cell cycle progression and cytokine signaling act in concert to govern peripheral Th1 cell fate.

**Conclusion:**

Our data indicates that DOCK2 acts as a protective role in regulating systemic inflammation and multi-organ injury in bacterial sepsis by constraining Th1 response. These findings provide new targets for immunomodulatory therapy of sepsis, suggesting that targeting the DOCK2-Th1 axis may become a new strategy to improve systemic inflammatory responses associated with bacterial infections.

## Introduction

Sepsis is a life-threatening organ dysfunction caused by a dysregulated host response to infection, which remains a major cause of global morbidity and mortality (1, 2). Septic shock, a subtype of sepsis that is characterized by severe cardiovascular abnormalities, is characterized by the more excessive production of cytokines and a higher risk of death (3). The integral Gram-negative bacteria wall component, lipopolysaccharide (LPS) can provoke life-threatening septic shock (4). Sepsis has become the main cause of death and critical disease worldwide (5). Hence, it is urgently required further studies to reveal the molecular and cellular mechanism of sepsis.

Sepsis is characterized by “cytokine storm,” which subsequently leads to overwhelming organ damage and mortality (4). Interferon-γ (IFN-γ) outstands one of the major proinflammatory cytokines in sepsis, as IFN-γ– or T cell–deficient mice are tolerant to polymicrobial sepsis and *Escherichia coli* (*E. coli*) infections, and neutralization of IFN-γ recovers mice from endotoxic shock (6, 7). T helper 1 (Th1) cell responses have been known as massive IFN-γ production and promoted the pathogenesis of sepsis (8). However, the underlying mechanisms remain largely unknown.

Dedicator of cytokinesis 2 (DOCK2), a member of the CDM family of proteins, is a guanine nucleotide exchange factor that is highly expressed in lymphoid related tissues including the bone marrow, spleen, and lymph nodes and selectively expressed in hematopoietic cells (9, 10). Additionally, DOCK2 is essential for the regulation of the immune system by affecting the adhesion, migration, proliferation, and differentiation of immune cells (10–12). DOCK2-deficient mice exhibit a severe reduction of plasmacytoid dendritic cells in the spleen and lymph nodes and selective loss of type I IFN induction (13). In addition, it was reported that biallelic *Dock2* mutations in human cause severe combined immunodeficiency with early-onset, invasive bacterial and viral infections (14). All these above highlighted the central role of DOCK2 in immune regulation in both humans and mice. Nevertheless, the regulatory role of DOCK2 on IFN-γ–producing Th1 response in LPS-induced sepsis has not yet been elucidated.

In this work, we identified an essential role for DOCK2 in bacterial sepsis. *Dock2^−/−^* mice have shown reduced animal survival after LPS-induced sepsis and exhibited much severe organ injury and more excessive Th1 immune response. Furthermore, DOCK2 deficiency promoted host susceptibility to *E. coli* sepsis and exhibited evaluated Th1 response that is required. Ulteriorly, depletion endogenous CD4^+^ T cells alleviated susceptibility of LPS-induced sepsis in *Dock2^−/−^* mice. Mechanistically, DOCK2-mediated cell cycle progression and cytokine signaling act in concert to govern peripheral Th1 cell fate. Overall, these findings suggest DOCK2 as an essential, negative regulator in LPS responses that protect the host from harmful hyperresponsiveness to LPS and may provide new insight into the endotoxin-induced sepsis.

## Materials and methods

### Mice

*Dock2^−/−^* mice and wild-type (WT) C57BL/6J mice were purchased from GemPharmatech Co., Ltd. (China). Sex-matched *Dock2^−/−^* mice and WT littermate controls were used at 8–12 weeks old (body weight, 20–25 g). All mice were maintained under specific pathogen–free conditions with restricted 12-h day/night cycle at a temperature 18°C–22°C and humidity 50%–60%. Protocols for animal experiments were approved by the Institutional Animal Care and Use Committees of the Southern Medical University. For body weights assays, mice that lost ≥ 20% of initial weight were euthanized.

### Mouse model of LPS-induced sepsis

*Dock2^−/−^* and WT mice were intraperitoneally (i.p.) injected with *E. coli* LPS (5 mg/kg or 25 mg/kg body weight, O55:B5, Sigma), diluted in pyrogen-free phosphate-buffered solution (PBS) or PBS as control. The survival, weight, and eye exudate formation of the mice were monitored over the next 72 h. Mice were euthanized, and the peripheral blood and the organs (spleen, lung and liver) were harvested at 72 h after LPS challenge and used in the assays described below.

### Mouse model of *E coli* sepsis

The *E. coli* strain (American Type Culture Collection (ATCC) 25922) was stored at −80°C in 50% glycerol. *E. coli* stock solution (10 μL) was incubated in 10 mL of Luria-Bertani (LB; Sangon Biotech) medium at 37°C and 200 Revolutions Per Minute (rpm) shaking for 12 h and washed twice with cold PBS. The optical density 600nm (OD_600_) (optical density 600nm) value was measured in a spectrophotometer to estimate the number of *E. coli* [OD_600_ = 1 = 2 × 10^9^ colony-forming units (CFU)/mL]. The bacterial density was adjusted to 1 × 10^9^ CFU/mL. *Dock2^−/−^* and WT mice were i.p. injected 2 × 10^8^ CFU of *E. coli* in 200 μL of PBS. Survival rates were monitored for 72 h.

### Determination of bacterial burden

To determine the bacterial burden in mice, blood and peritoneal lavage fluid (PLF) of mice were obtained 72 h after *E. coli* injection. Blood and PLF were serially diluted with sterile PBS. Serial dilutions (50 μL) were seeded on LB agar plates and incubated at 37°C for 12–18 h to determine the CFU of *E. coli*. The data were log-transformed for statistical analysis.

### *In vivo* neutralization experiments

For the neutralization of IFN-γ experiments, mice were i.p. treated with 300 μg of anti–IFN-γ–InVivo (XMG1.2, Selleck) as well as isotype controls (rat IgG2b isotype control–InVivo, Selleck) 4 h after LPS injection and then monitored survival for 72 h. For the CD4^+^ T-cell depletion experiments, mice were received anti–CD4-InVivo (GK1.5, Selleck) (i.p., 200 μg per mouse every 2 days) at the day before LPS injection.

### Preparation of single-cell suspensions from mouse tissue samples

After anesthetizing mice with isoflurane, the peripheral blood of the mice was collected into a heparin anticoagulant tube, then euthanized, and perfused with cold PBS through the right ventricle of the heart before removal of tissues. For lung and liver, tissues were weighed, and dissected tissues were cut into small pieces and digested for 45 min at 37°C with collagenase type D (1 mg/mL; Worthington) and deoxyribonuclease (DNase) I(25 μg/mL; Roche) in Roswell Park Memorial Institute 1640 medium (RPMI 1640) medium supplemented with 10% Fetal Bovine Serum (FBS) (Biological Industries) and 1% penicillin/streptomycin (Life Technologies). Digested samples were passed through 70-μm cell strainers (BD Falcon) and washed with flow staining (Fluorescence-activated cell sorting (FACS)) buffer [PBS containing 1% FBS and 2 mM Ethylene Diamine Tetraacetic Acid (EDTA) (pH 8.0)]. Mononuclear leukocytes were fractionated by a 40%–80% (lung) or 30%–70% (liver) Percoll (GE Healthcare) density gradient centrifugation and washed in FACS buffer. Then, red blood cells (RBCs) were lysed in 1× ammonium-chloride-potassium (ACK) buffer. Single-cell suspensions were used for subsequent flow cytometry staining. Suspensions of spleen cells were obtained by mashing the spleen through a 70-mm nylon cell strainer, and RBCs were lysed in 1× ACK buffer. For peripheral blood serum isolation, peripheral blood was left to settle at 4°C until serum precipitation occurred and centrifugated at 1,200g for 7 min at 4°C to obtain serum for cytokine concentration detection.

### Flow cytometry analysis and sorting

Single-cell suspensions were blocked with anti-Fc receptor blocking antibody (anti-CD16/CD32, BioLegend, clone 93) in FACS buffer for 10 min to avoid false-positive staining.

For surface protein of cells staining, single-cell suspensions were counted and diluted into proper concentrations and incubated for 30 min in the dark at 4°C with the following fluorescently conjugated antibodies: CD3 (eBioscience, clone 17A2), CD4 (eBioscience, clone GK1.5), CD45 (BioLegend, clone 30-F11), NK1.1 (eBioscience, clone PK136), CD11c (eBioscience, clone N418), CD11b (eBioscience, clone M1/70), F4/80 (eBioscience, clone BM8), CD8a (eBioscience, clone 53-6.7), Ly6C (eBioscience, clone HK1.4), and Ly6G (eBioscience, clone RB6-8C5). The stained cells were then washed with FACS buffer and further stained with Fixable Viability Stain 700 (BD Biosciences) at 4°C under darkness for 30 min.

For detection of intracellular cytokine expression, the stained cells were suspended and stimulated in complete RPMI 1640 medium + 10% FBS with Phorbol 12-myristate 13-acetate (PMA) (50 ng/mL; Sigma-Aldrich), ionomycin (1 μg/mL; Sigma-Aldrich), and brefeldin A (1 μg/mL; eBioscience) for 4 h at 37°C and 5% CO_2_. Cells were subsequently surface protein-stained, fixed (intracellular fixation buffer, eBioscience), permeabilized (10× permeabilization buffer, eBioscience), and stained with indicated cytokine antibodies: IFN-γ (BioLegend, clone XMG1.2), and tumor necrosis factor–α (TNF-α; BioLegend, clone MP6-X722). Ki-67 (BioLegend, clone 16A8) staining was performed with the Foxp3/Transcription Factor Staining Buffer Set (Thermo Fisher Scientific) according to the manufacturer’s instructions. Various immune cells of mouse lung, liver, spleen, and Peripheral blood (PB) were gated as Th1 cells (Live&Dead^−^CD3^+^CD4^+^IFN-γ^+^), CD8^+^ T cells (Live&Dead^−^ CD8^+^), and macrophage cells (Live&Dead^−^CD11b^+^ F4/80^+^). All stained cells were acquired on an LSRFortessa flow cytometer (BD Biosciences) and analyzed by FlowJo V10.0.7.

For flow cytometric sorting, CD4^+^ T cells of spleen were sorted from *Dock2^−/−^* and WT mice at sepsis state for RNA sequencing (RNA-seq). Firstly, debris and doublets were excluded for cell types using forward scatter (FSC) and side scatter (SSC). Then, CD4^+^ T cell was gated as follow: CD44^+^CD4^+^ T cells (Live&Dead^−^CD4^+^CD44^+^). BD FACSAria III Cell Sorter (BD Biosciences) was used. The gating strategies and sorting purity of flow cytometry were included in the supplemental information.

### Histopathological analysis

The spleen, lung, and liver tissues were perfused with cold PBS to reduce blood cell-induced background and then were harvested and fixed in 4% phosphate-buffered formaldehyde solution for at least 24 h. Fixed and paraffin-embedded tissues were cut into 5-μm-thick sections, followed by hematoxylin and eosin (H&E) staining, and tissue injury severity analysis was measured under a microscope (Olympus BX63).

### ELISA

The levels of mouse IFN-γ, TNF-α, interleukin-6 (IL-6), and IL-1β in serum were measured by enzyme-linked immunosorbent assay (ELISA) according to the manufacturer’s protocol (R&D). Serum was prepared from blood collected from the eye socket after LPS treatment. After centrifugation, the supernatants were used for cytokine measurement.

### Bulk RNA-seq and data analysis

Five thousand activated CD4^+^ T cells (Live&Dead^−^CD3^+^CD4^+^CD44^hi^) from the spleen of LPS challenged mice were lysed in 10 μL of TCL buffer plus 1% 2-mercaptoethanol. Libraries were processed with SMARTSeq2 (15) with three biological replicates per condition and paired-end sequenced (150 bp × 2) with a 75-cycle NextSeq 500 high-output V2 kit. Obtained RNA-seq reads were aligned to the mouse genome and transcriptome (GENCODE GRCm38 vM25, mm10) using Spliced Transcripts Alignment to a Reference (STAR) (version 2.7.5c) (16) with “twopassMode Basic,” expression abundances were estimated using RSEM (version 1.3.3) (17). The count matrices’ outputs from gene results were processed with the R package (version 3.32.0) to analyze differential gene expression (18) with default parameters. Kyoto Encyclopedia of Genes and Genomes (KEGG) enrichment was performed using custom script with clusterProfiler (version 3.16.1) (19) and hierarchical file from “http://rest.kegg.jp” and visualized using ggplot2 (version 3.3.3) (20). Gene set enrichment analysis (GSEA) was run with the official tool (GSEA version 4.2.1, MsigDB hallmark v7.3) and replot in R. Overall expression was computed as previously described (21, 22).

### Statistical analysis

Data analysis and representation were performed with Prism (GraphPad version 10.0). The *p*-values were calculated using an unpaired two-tailed Student’s t-test or two-way ANOVA. A *p*-value < 0.05 was considered statistically significant and are displayed as follows: *p < 0.05; **p < 0.01; ***p < 0.001; ****p < 0.0001; and not significant (ns), *p* > 0.05. Data in all figures are displayed as mean ± SEM.

## Results

### DOCK2-deficient mice are more hyperresponsive to LPS

**Figure 1:**
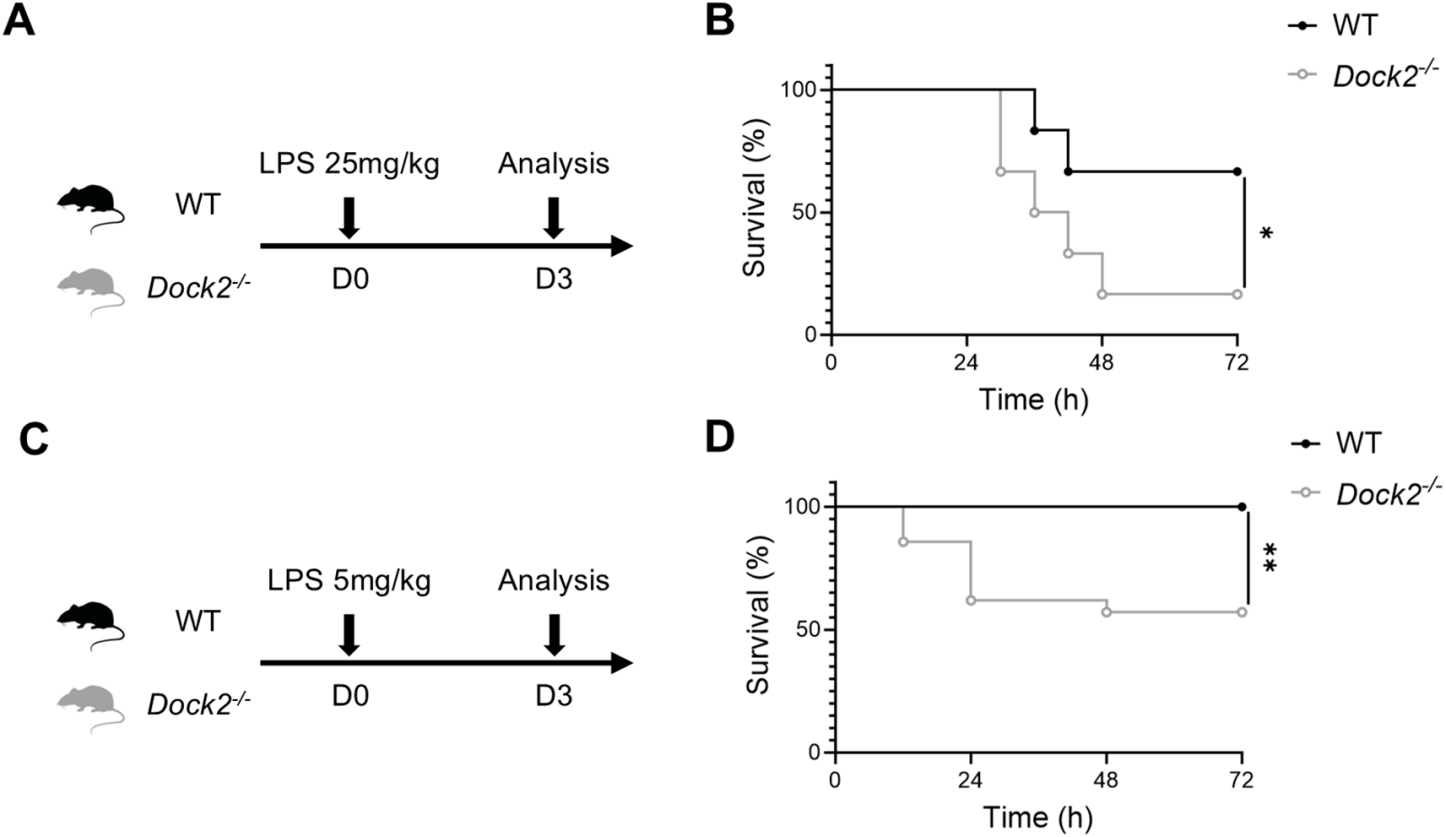
Reduced survival in DOCK2-deficient (*Dock2^−/−^*) mice after LPS challenge. **(A–D)** Eight to 12-week-old WT and *Dock2^−/−^* (n ≥ 12 per group) mice were intraperitoneally (i.p.) injected with LPS (25 mg/kg or 5 mg/kg body weight), and survival was monitored. **(A, C)** Experimental design. **(B, D)** The survival rate of septic mice expressed as a percentage. *p < 0.05 and **p < 0.01 by Kaplan–Meier analysis.

To investigate the involvement of DOCK2 in the sensitivity to bacterial pathogen, we i.p. injected various doses of *E. coli* LPS into age- and sex-matched *Dock2^−/−^* mice and WT cohorts (**Figures 1A, C**). Almost all *Dock2^−/−^* mice but not WT littermates died within 72 h after challenge with 25 mg/kg body weight LPS (**Figure 1B**). Then, we narrowed down LPS dose to 5 mg/kg body weight as shown in **Figure 1C** and found that nearly half of *Dock2^−/−^* mice still died within 72 h after LPS administration, whereas it does not cause mortality in the WT littermates (**Figure 1D**). These data led to the notion that DOCK2 plays a protective role in LPS-induced sepsis.

### DOCK2-deficient mice suffer more severe endotoxin-induced septic shock

**Figure 2:**
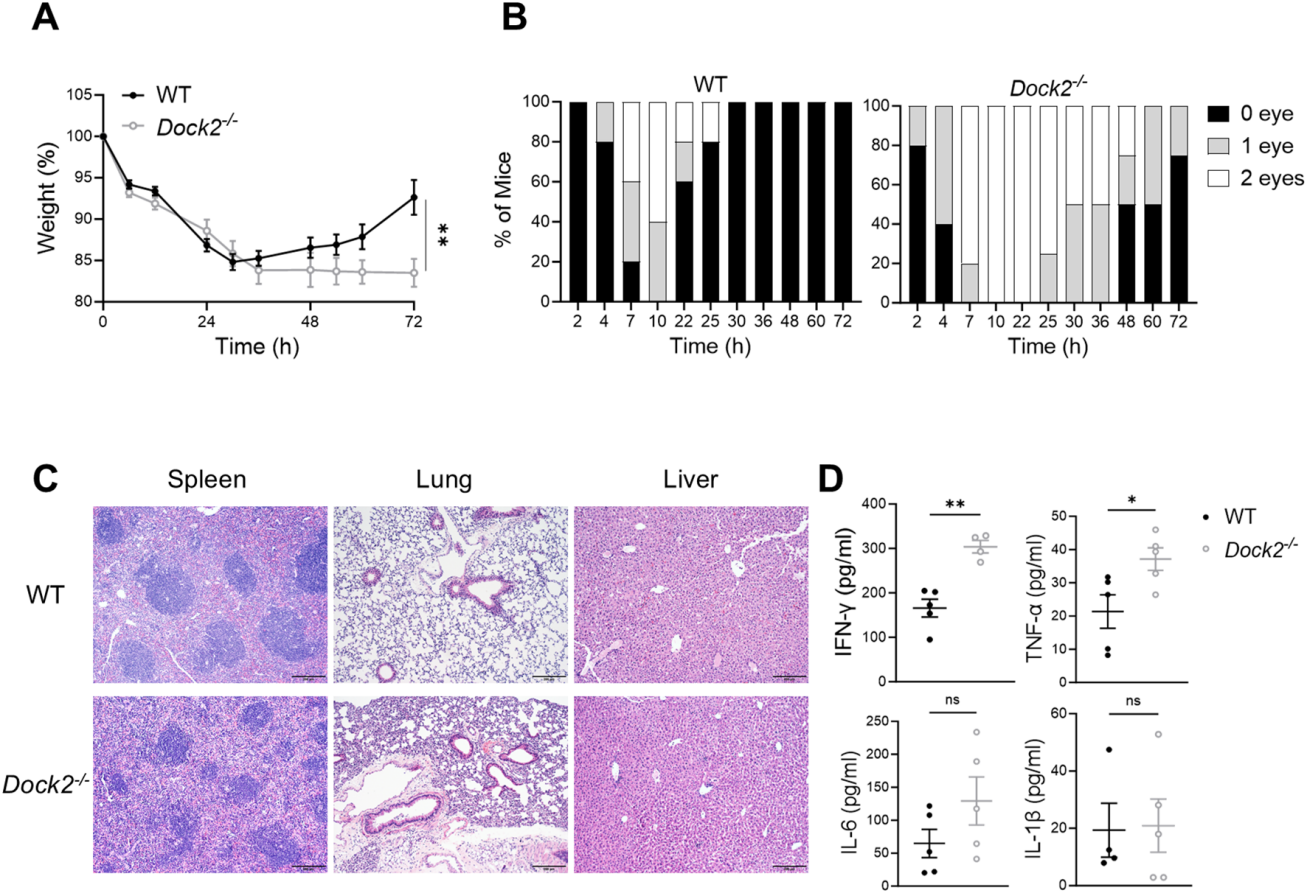
*Dock2^−/−^* mice are more susceptible to LPS-induced sepsis. **(A–D)** *Dock2^−/−^* mice and WT littermates (n ≥ 4 per group) i.p. injected with LPS (5 mg/kg) as in **Figure 1C**; weight loss was recorded as a measure of sickness and recovery; and eye exudate formation in zero, one, or two eyes were monitored up to 72 h. On 72 h after LPS administration, mice were anesthetized, and tissues were harvested. Weight loss curves **(A)** and eye exudate formation **(B)** for mice after LPS administration. **(C)** Representative morphological changes of the spleen, lung, and liver section from septic mice (H&E staining, ×100 magnification). **(D)** The serum concentrations of IFN-γ, TNF-α, IL-6, and IL-1β in *Dock2^−/−^* mice were measured at 72 h after LPS injection. For all panels, error bars show the means ± SEM. Not significant (ns), *p < 0.05; **p < 0.01 by an unpaired Student’s t-test. Data are representative of three independent experiments.

The weight loss and eye exudate formation of mice in sepsis induced by systemic inflammation can provide references for sickness progression and recovery. Thus, we tracked weight loss and eye exudates in septic mice administrated with LPS (5 mg/kg of body weight). The weight loss in *Dock2^−/−^* mice and WT littermate controls was comparable within 36 h. As time went on, the weight of WT mice but not *Dock2^−/−^* ones began to recover. At 72 h after LPS injection, *Dock2^−/−^* mice showed lower body weight when compared to WT littermates (**Figure 2A**). In addition, DOCK2 deficiency led to more eye exudate formation and blunted recovery of eye exudates (**Figure 2B**). As sepsis is closely associated with multiple-organ injury and dysfunction, we next evaluated histopathology changes in the spleen, lung, and liver of the *Dock2^−/−^* and WT mice by H&E staining. The *Dock2^−/−^* mice exhibited structural disorganization in the spleen, severe injury and inflammation in the lung, thicken alveolar wall and inflammatory cell infiltration increasing in the lung, and widespread hemorrhaging in the liver, whereas the WT ones had much slighter organ damage after LPS treatment (**Figure 2C**). Increased serum aspartate aminotransferase (AST) also indicated more severe sepsis-induced liver injury in *Dock2^−/−^* mice (**Supplementary Figure 2**). As sepsis is characterized by “cytokine storm” which subsequently leads to overwhelming organ damage and mortality (4). We next focused on proinflammatory cytokines that play a major role in sepsis, including IFN-γ, TNFα, IL-6, and IL-1β. Serum cytokine levels were determined at 72 h after LPS treatment. As the data shown, the production of major proinflammatory cytokines, including IFN-γ and TNF-α, were significantly evaluated in *Dock2^−/−^* mice compared with WT ones (**Figure 2D**). Together, these data suggest that DOCK2-deficiency lead to more susceptible to LPS-induced sepsis.

### Enhanced IFN-γ–producing CD4^+^ T cells in septic *Dock2^−/−^* mice

**Figure 3:**
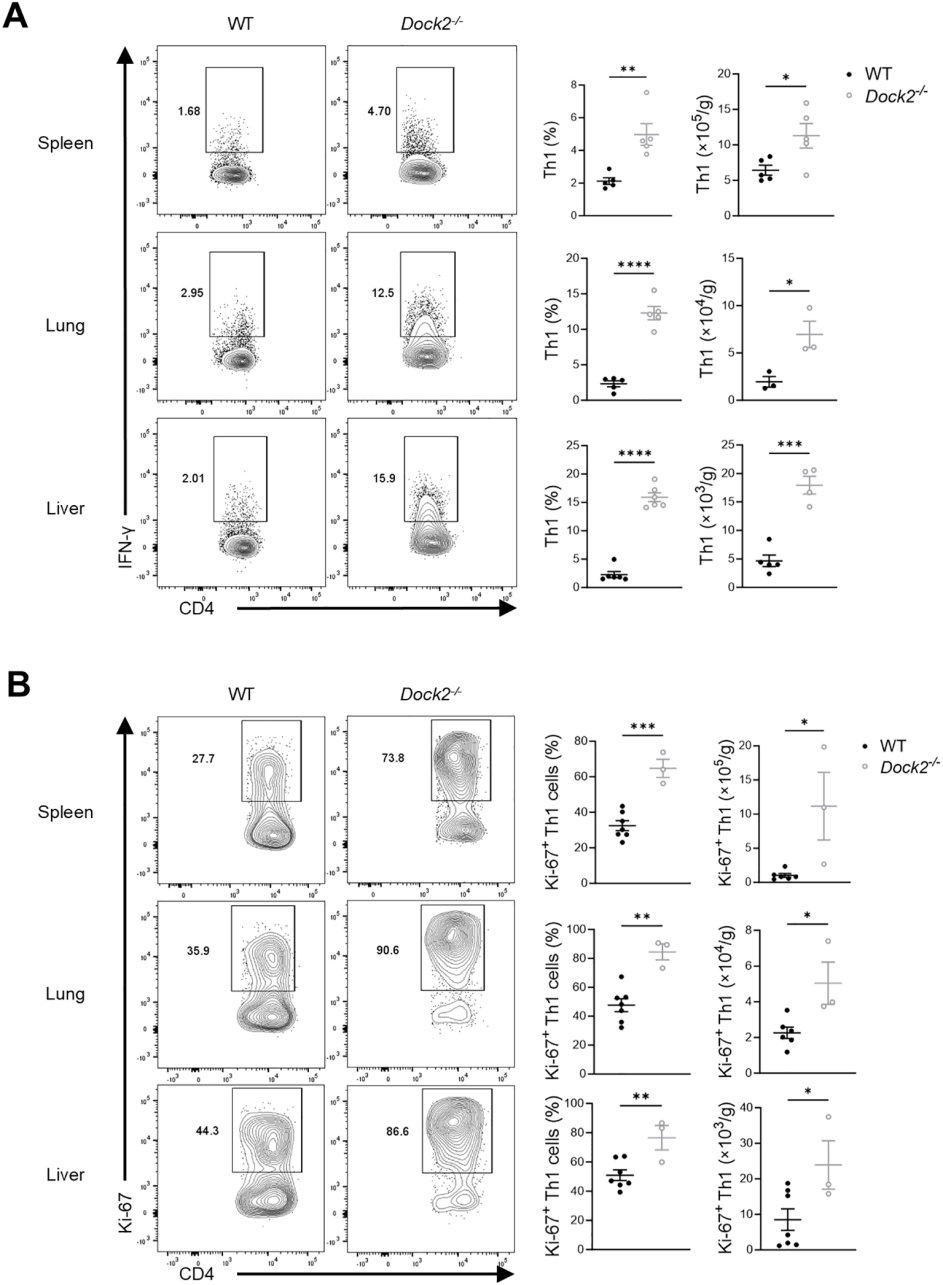
*Dock2^−/−^* mice exhibit an enhanced Th1 response to LPS. **(A, B)** *Dock2^−/−^* mice and WT littermates (n ≥ 3 per group) i.p. injected with 5 mg/kg of LPS as in **Figure 1C**. On day 3 after injection, mice were anesthetized and peripheral blood, spleen, lung, and liver were harvested, and immune cells were isolated for flow cytometric analysis. **(A)** Based on the gating strategies shown in **Supplementary Figure 1**. Representative flow plots of Th1 cells (Live&Dead^−^CD3^+^CD4^+^IFN-γ^+^) and statistical analysis of percentages and relative numbers of Th1 cells are shown. **(B)** Ki-67^+^ Th1 cells were further analyzed. Representative flow plots and statistical analysis of the percentages and relative numbers of Ki-67^+^ Th1 cells are shown. For all panels, error bars show the means ± SEM. *p < 0.05, **p < 0.01, ***p < 0.001, and ****p < 0.0001 by an unpaired Student’s t-test. Data are representative of three independent experiments.

Sepsis is characterized by concurrent unbalanced hyperinflammation and aberrant immune responses. To explore the immune mechanisms by which *Dock2^−/−^* mice exhibited much higher level of IFN-γ in the LPS-induced sepsis, we next profiled the major potential immune cells closely associated with sepsis, including macrophages (23), nature killer (NK) cells (24), and T cells (25). Flow cytometry analyzes that the loss of DOCK2 had increased frequencies and numbers of IFN-γ–producing CD4^+^ T cells (also named Th1) in the spleen, lung, and liver compared with those in control mice (**Figure 3A**). Consistently, we found that *Dock2^−/−^* mice exhibited higher frequencies of Ki-67–expressing Th1 cells than WT littermate controls (**Figure 3B**). However, DOCK2 deficiency had minor role in regulating macrophages as well as IFN-γ–producing CD8^+^ T and NK cells in the above tissues from LPS-induced septic mice (**Supplementary Figures 3A–C**). Given that IL-12 was the major driver of IFN-γ production and Th1 polarization, we observed markedly increased serum IL-12 in *Dock2* ^−/−^ mice at 12 h after LPS injection (**Supplementary Figure 4A**). Consistently, Th1 cells also elevated in in *Dock2* ^−/−^ mice at 12 h after LPS treatment (**Supplementary Figure 4B**). Moreover, we also investigated the expression profile of DOCK2 in Th1 cells at different time points. The data revealed a downward expression of DOCK2 after LPS treatment and a much lower DOCK2 expression at 72 h after LPS injection, indicating that DOCK2 has an important role in the host immune response to septic inflammation (**Supplementary Figure 4C**). Thereafter, we mainly focus on 72 h after sepsis induction. Further analysis showed that *Dock2^−/−^* and WT control mice also manifested comparable frequencies of TNF-α–producing CD4^+^ T, CD8^+^ T, and NK cells and macrophages (**Supplementary Figures 5A–D**). Collectively, hyperresponsive IFN-γ–producing CD4^+^ T cells in septic *Dock2^−/−^* mice might contribute to the severe inflammation and organ injury.

### 2 DOCKdeficiency causes high susceptibility to *E. coli* sepsis and overwhelming Th1 response

**Figure 4:**
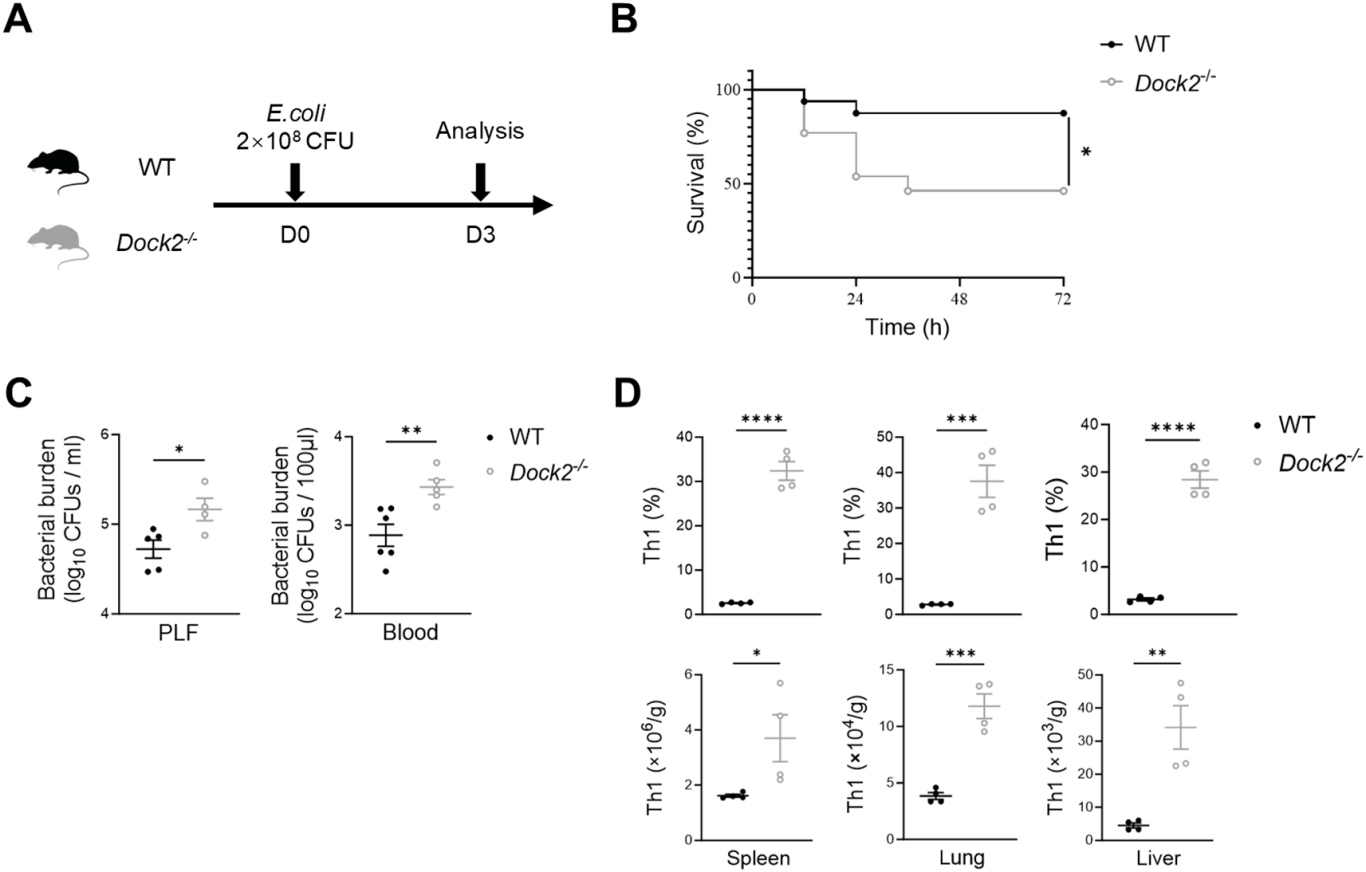
Deficiency of DOCK2 enhances host susceptibility and Th1 response to *E. coli* sepsis. **(A–D)** Eight- to 12-week-old WT and *Dock2^−/−^* mice were i.p. injected with *E. coli* (2 × 10^8^ CFU), and survival was monitored for 72 h. Seventy-two hours after *E. coli* administration, mice were anesthetized, and tissues were harvested. **(A)** Experimental scheme. **(B)** The survival rate of *E. coli* septic mice expressed as a percentage (n = 13 for *Dock2^−/−^* mice and n = 16 for WT mice). **(C)** The bacterial burden in the blood and PLF 72 h after injection of *E. coli* was determined (n ≥ 4 per group). **(D)** Based on the gating strategies shown in **Supplementary Figure 1**, statistical analysis of percentages and relative numbers of Th1 cells (Live&Dead^−^CD3^+^CD4^+^ IFN-γ^+^) are shown (n = 4 per group). For all panels, error bars show the means ± SEM. *p < 0.05, **p < 0.01, ***p < 0.001, and ****p < 0.0001 by Kaplan–Meier analysis for survival rate and an unpaired Student’s t-test for others. Data are representative of three independent experiments.

Although DOCK2 protected against LPS-induced shock via blunting the cytokine storm, especially IFN-γ response, the role of DOCK2 in resisting live bacterial infections remains largely unknown. To address this issue, we studied *E. coli* sepsis. Namely, *Dock2^−/−^* mice and WT littermates were i.p. injected with *E. coli* ATCC25922 (**Figure 4A**). Expectedly, we observed impaired survival of *Dock2^−/−^* mice when compared to with WT animals within 72 h of *E. coli* infection (**Figure 4B**). This was paralleled by a substantial increase in the bacterial burden in PLF and peripheral blood of *Dock2^−/−^* mice (**Figure 4C**). In addition, we found that the deficiency of DOCK2 significantly augmented the frequencies and numbers of CD4^+^IFN-γ^+^ inflammatory Th1 cells in the spleens, lungs, and livers in response to *E. coli* infection (**Figure 4D**), resembling the phenotypes of LPS challenge. Taken together, these data indicated that DOCK2 provides robust protection against live bacterial infection, other than LPS-induced sepsis.

### Blockade of IFN-γ–producing CD4^+^ T cells alleviates LPS-induced sepsis on *Dock2^−/−^* mice

**Figure 5:**
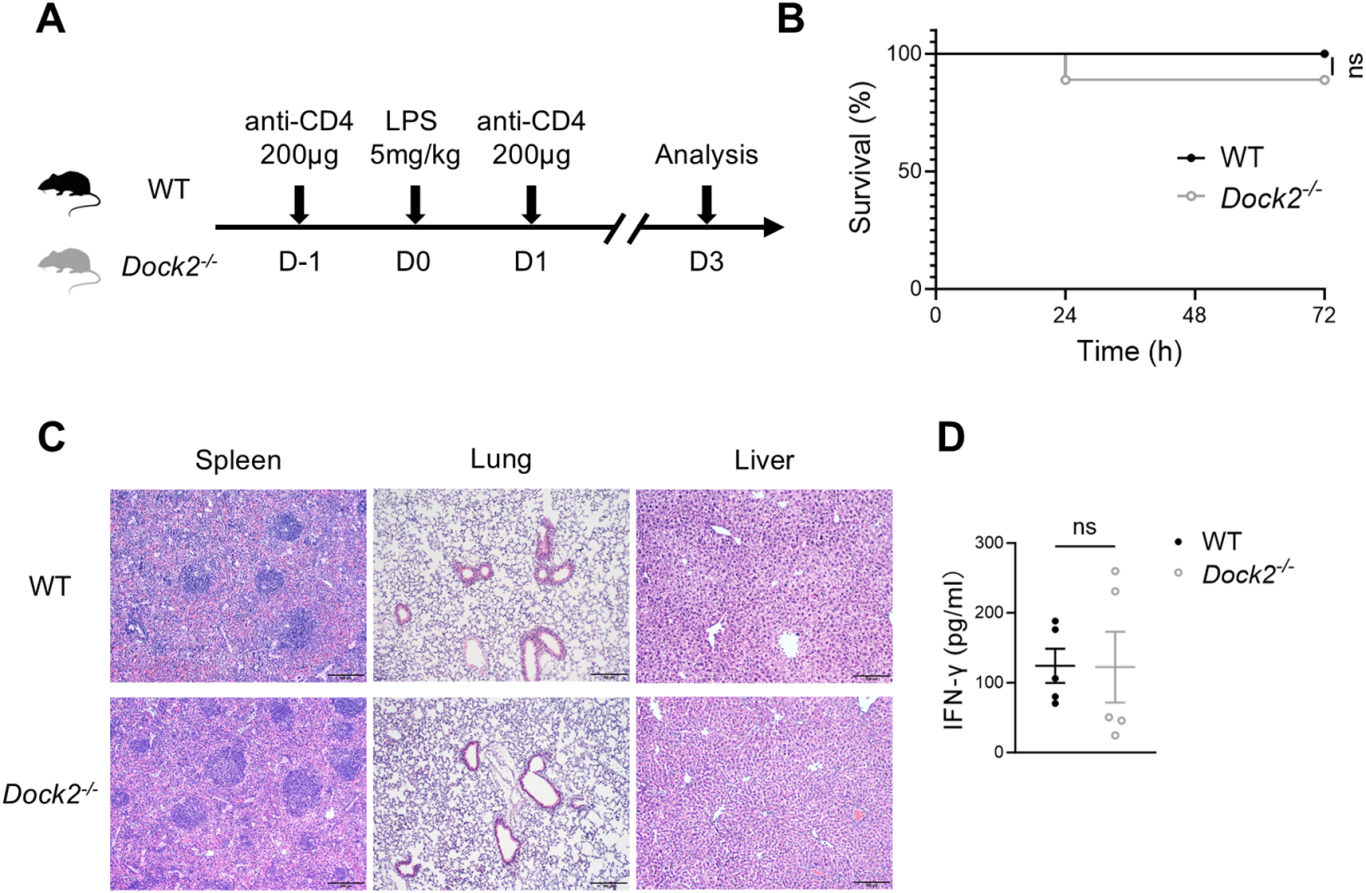
CD4^+^ T-cell depletion alleviates the effects of LPS-induced sepsis on DOCK2-deficient mice. **(A–D)** *Dock2^−/−^* mice and WT littermates i.p. injected with anti-CD4 antibody (200 μg per mice per time) or isotype controls and LPS (5 mg/kg of body weight). Survival of mice was monitored for 72 h. Seventy-two hours after LPS administration, mice were anesthetized, and tissues were harvested. **(A)** Experimental scheme. **(B)** The survival rate of LPS-induced septic mice expressed as a percentage (n = 9 per group). **(C)** Representative morphological changes of the spleen, lung, and liver section from septic mice (H&E staining, ×100 magnification). **(D)** The serum concentrations of IFN-γ were measured at 72 h after LPS injection. Not significant (ns), by Kaplan–Meier analysis for survival rate and an unpaired Student’s t-test for IFN-γ level. Data are representative of three independent experiments.

To further explore whether DOCK2 regulates IFN-γ–producing CD4^+^ T cells that trigger LPS-induced sepsis or not, firstly, we detected the role of IFN-γ in DOCK2-deficient mice when they suffered from endotoxin-induced septic shock. *Dock2^−/−^* mice were i.p. administered anti–IFN-γ or PBS 4 h after LPS injection and then monitored survival for 72 h. Data show that blocking IFN-γ efficiently prolonged the survival of LPS-treated *Dock2^−/−^* mice (**Supplementary Figures 6A, B**). These data suggested IFN-γ played a critical role in process of endotoxin-induced septic shock in *Dock2^−/−^* mice. Furthermore, we i.p. treated *Dock2^−/−^* mice and WT littermates with depleting anti-CD4 antibody (26, 27) ahead of LPS administration and strengthen depletion at on the third day, and survival was monitored (**Figure 5A**). As shown in **Figure 5B**, the depletion of CD4^+^ T cells significantly increased the survival rate of *Dock2^−/−^* mice within 72 h after LPS injection, and there was basically no death. At day 3 after LPS treatment, we confirmed the depletion efficiency of anti-CD4 antibody by flow cytometric analysis of CD4^+^ T cells. As expected, anti-CD4 antibody treatment resulted in the clearance of CD4^+^ T cells from the spleen, lung, and liver (**Supplementary Figures 6C, D**). Moreover, histomorphologic analysis of the spleen, lung, and liver showed that, compared with WT littermate controls, *Dock2^−/−^* mice had no obvious structural disorganization in the spleen and no obvious infiltration of inflammatory cell in liver and lung tissue (**Figure 5C**). Consistently, CD4^+^ T cell–depleting *Dock2^−/−^* mice manifested comparable serum IFN-γ, when compared with CD4^+^ T cell–depleting WT controls (**Figure 5D**). All these results together suggested that IFN-γ–producing CD4^+^ T cells are critical for systemic inflammation and multiple-organ injury in LPS-induced septic DOCK2-null mice.

### 1 DOCK2-mediated cell cycle progression and cytokine signaling act in concert to govern peripheral Thcell fate

**Figure 6:**
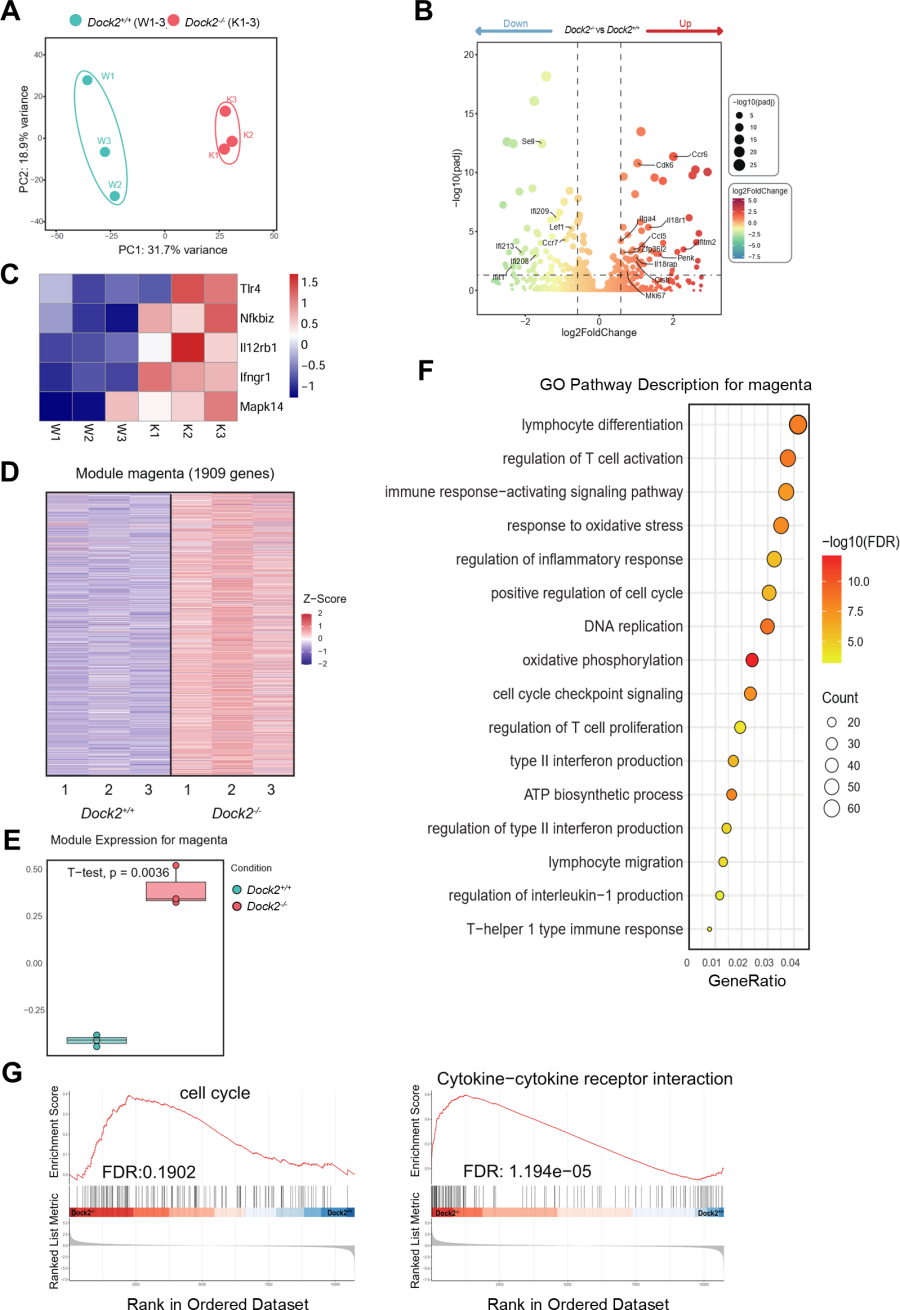
DOCK2 is required for cell cycle progression of peripheral Th1 cells. **(A–F)** Bulk RNA-seq analysis of spleen activated T cells (CD3^+^CD4^+^CD44^+^) from DOCK2-deficient mice and WT littermates (n = 3 per group). **(A)** Principal component analysis plot based on the top 2,000 most variable genes. **(B)** Volcano plot from DESeq2 analysis depicting differential expressed genes (DEGs), with significant downregulated genes marked in green and upregulated genes in red. Key genes are additionally highlighted. **(C)** Upregulated TLR and IFN-γ signaling genes in DOCK2-deficient mice. **(D–F)** Weighted gene co-expression network analysis depicting the magenta module, which contains 1,909 genes and is significantly enriched in *Dock2^−/−^* mice **(D)** Heatmap of normalized expression levels (Z-scores) of genes in magenta module, comparing *Dock2^+/+^* and *Dock2^−/−^* conditions. **(E)** Comparative expression levels of the magenta module between *Dock2^+/+^* and *Dock2^−/−^* mice, with statistical significance indicated (unpaired two-tailed Student’s t-test). **(F)** Dot plot of the Gene Ontology (GO) enrichment results for the 1,909 genes in the magenta module, with the x-axis representing gene ratio and with the dot color and size indicating the −log10 of the adjusted *p*-value and count of enriched genes, respectively. Key GO biological pathways are annotated. **(G)** Gene set enrichment analysis (GSEA) of hallmark 10,731 filtered genes with indicated false discovery rate (FDR).

To investigate the mechanisms underlying DOCK2-mediated suppression of Th1 responses in the septic mouse, we performed bulk RNA-seq analysis on CD3^+^CD4^+^ CD44^+^ T cells sorted from WT and *Dock2^−/−^* mice treated with LPS. Principal component analysis clearly segregated the activated CD4^+^ T-cell transcriptome profile of *Dock2^−/−^* mice from that of WT controls, suggesting the populations to be transcriptionally distinct (**Figure 6A**). Differentially expressed gene (DEG) analysis revealed that *Dock2^−/−^* CD4^+^ T cells expressed higher levels of cell cycle genes (*Cdk6*) (28), proliferation genes (*Mki67*), IFN-induced genes (*Ifitm2*) (29), cytokine receptor genes (*Il1r2*, *Il18r1*, *Il7r*, and *Il18rap*) (30), gene associated with migration and adhesion (*Ccr6*) (31), T-cell migration gene (*Itga4*) (32), immune cell homing gene (*Ccl5*) (33), and immunoregulatory gene (*Lilrb4a*, *Zfp36l2*, *Penk*, and *Cish*) (34, 35) compared to *Dock2^+/+^* CD4^+^ T cells (**Figure 6B**). More significantly, DOCK2 deficiency resulted in upregulated TLR and IFN-γ signaling genes, including Tlr4, Nfkbi2, Il12rb2, Ifngr1, and Mapk14 (**Figure 6C**). Further weighted gene co-expression network analysis was performed on the DEGs to identify co expression modules (**Figures 6D, E**). Gene Ontology analysis demonstrated that the DEGs were mainly linked to lymphocyte differentiation and regulation of T-cell activation (**Figure 6F**). In addition, GSEA revealed that the cell cycle and cytokine-cytokine receptor interaction gene sets were differentially enriched between WT and *Dock2^−/−^* CD4^+^ T cells (**Figure 6G**). Thus, DOCK2 directly regulated the expression of its target genes involved in mitotic cell cycle and effector cytokine expression associated with the CD4^+^ T cells to control overwhelming Th1 responses.

## Discussion

Uncontrolled systemic inflammatory response and multiple-organ injury are thought to play a crucial role in the pathogenesis of sepsis (1, 3, 4). In this study, our data revealed that DOCK2-deficient mice had reduced survival rate in LPS-induced sepsis and were more susceptible to LPS-induced sepsis, including weight loss, eye exudate formation, and histomorphologic changes of various organs. These findings are somewhat different from a previous study by Xu and colleagues (36), which showed that 4-[3′-(2″-chlorophenyl)-2′-propen-1′-ylidene]-1-phenyl-3,5-pyrazolidinedione (CPYPP), a small-molecule inhibitor of DOCK2, alleviated the severity of endotoxemia-induced acute lung injury by inhibiting LPS-induced macrophage activation. It is known that macrophages, as a kind of innate immune cells, first trigger the production of various initial proinflammatory cytokines, which, in turn, activate T cells or NK cells to release IFN-γ in response to LPS, which might play an immune role in the early stage of disease. Nevertheless, we verified that DOCK2 plays a vital role in constraining IFN-γ–producing CD4^+^ T cells and protects against LPS-induced systemic inflammation and organ injury.

Prior studies have confirmed that biallelic mutations in DOCK2 impair T-cell activation (14), and patients with DOCK2 deficiency have T-cell mitochondrial dysfunction and lymphopenia (37). It has also been reported that T cell– and IFN-γ–deficient mice showed apparently reduced serum IL-6 levels and markedly resist *E. coli* infection or sepsis, indicating that IFN-γ–producing T cells play a vital role in bacterial sepsis (6, 7). Here, we explored the role of DOCK2 on Th1 immune response in mice during LPS-induced sepsis. Our data showed that DOCK2 deficiency enhanced Th1 cell immune response and proliferative capacity in LPS-induced sepsis. In addition, studies have pointed out that IFN-γ produced by Th1 cells has an important role in activating macrophages to increase their microbicidal activity (38). However, we compared macrophages in DOCK2-deficient and CK2-sufficient mice upon LPS treatment. There was a minor difference between *Dock2^−/−^* mice and their cohorts. Hyperresponsive IFN-γ–producing CD4^+^ T cells in septic *Dock2^−/−^* mice seemed independent of macrophages.

For further confirmation, we ascertain the effect of Th1 on DOCK2-deficient mice in LPS-induced sepsis by blocking IFN-γ and depleting CD4^+^ T cells *in vivo*. IFN-γ neutralization evidently prolonged the survival of LPS-treated *Dock2^−/−^* mice. Moreover, CD4^+^ T-cell depletion could obviously alleviate the mortality systemic inflammation and multiple-organ damage of LPS-treated DOCK2-deficient mice. Given the limitations of the LPS-induced sepsis model, we performed studies in *Dock2^−/−^* mice using clinically relevant live *E. coli* infection and identified that DOCK2 deficiency also promoted susceptibility and Th1 response to *E. coli* sepsis. Collectively, our study highlights DOCK2 potentially a protective target for sepsis intervention in mice.

Little is known about the mechanisms underlying DOCK2-mediated suppression of Th1 responses in the septic mouse, and we performed bulk RNA-seq analysis on activated CD4^+^ T cells sorted from *Dock2^+/+^* and *Dock2^−/−^* mice treated with LPS. We found that an obvious enrichment in gene sets associated with cell cycle progression and cytokine signaling governing peripheral Th1 cell fate is upregulated in *Dock2^−/−^* CD44^+^CD4^+^ T cells when compared to WT CD44^+^CD4^+^ T cells. The dedicated role of DOCK2 in regulation of Th1 response under different condition needs further studies in future.

## Data Availability

The original contributions presented in the study are publicly available. This data can be found here: NCBI GEO repository, accession number GSE297913.
