# Supplementary material for: DOCK2 protects against bacterial sepsis by constraining T helper 1 response
Source: Front Immunol. 2025 May 29;16:1527934. doi: 10.3389/fimmu.2025.1527934 (PMC12158923; doi:10.3389/fimmu.2025.1527934)
Supplement: Supplementary file 1 [file DataSheet1.pdf]

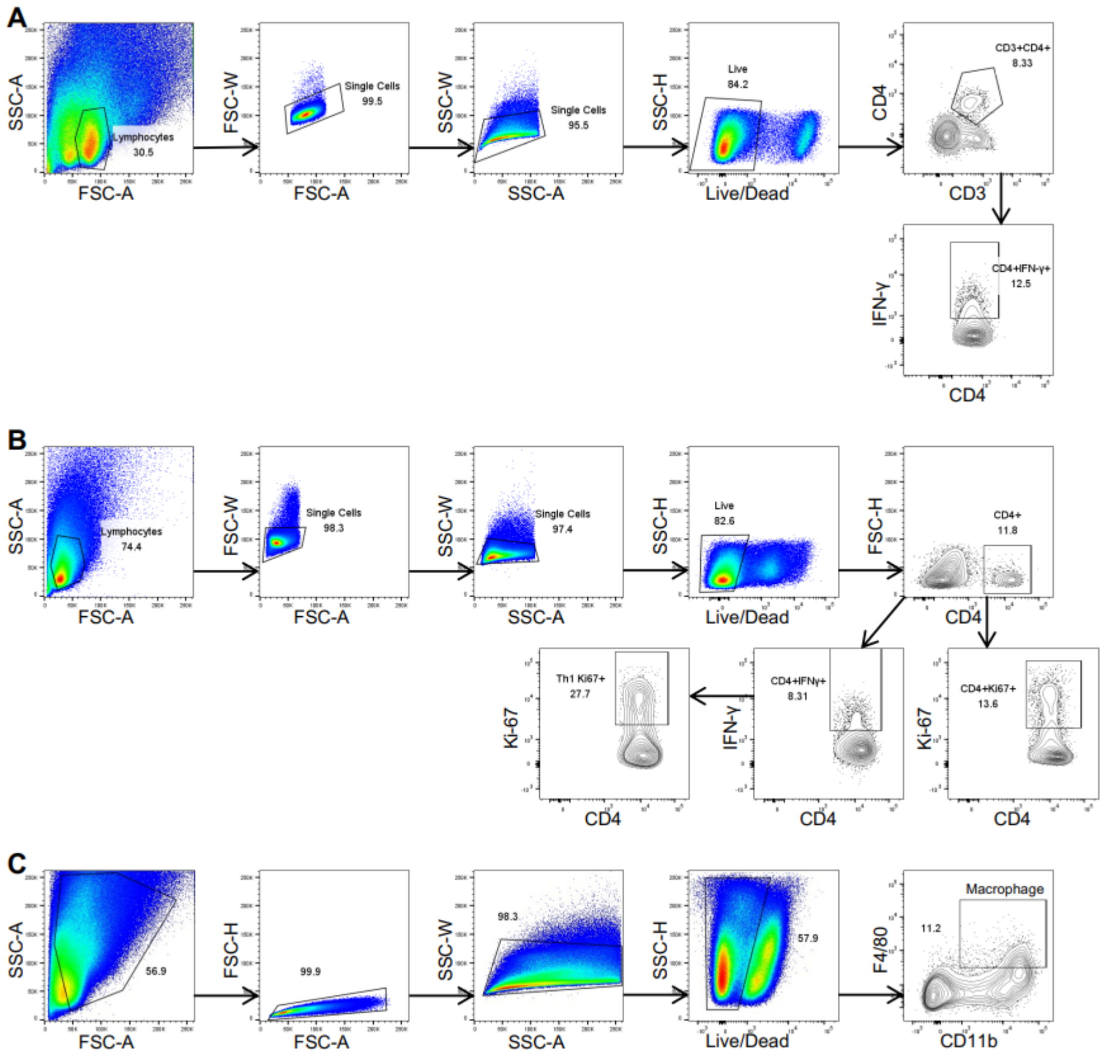

Supplemental Figure 1. The gating strategy for Th1 cells, Ki-67$^+$ Th1 cells, and Macrophages in various tissues. (A) Th1: Live&Dead$^-$CD3$^+$CD4$^+$IFN-γ$^+$. (B) Ki67$^+$ Th1: Live&Dead$^-$CD3$^+$CD4$^+$IFN-γ$^+$Ki-67$^+$. (C) Macrophages: Live&Dead$^-$CD11b$^+$ F4/80$^+$.

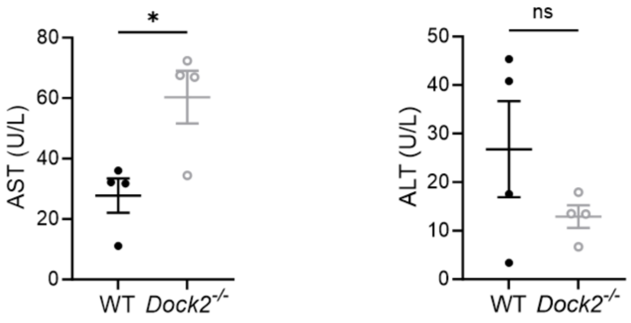

Supplemental Figure 2. Serum markers of liver dysfunction in WT and *Dock2$^{-/-}$* mice at 72 h after LPS challenge. *Dock2$^{-/-}$* mice and WT littermates (n ⩾ 4/group) i.p. injected with 5 mg/kg of LPS as in Fig.1C. On day 3 after injection, mice were anesthetized and serum were harvested for analysis. The serum concentrations of AST(aspartate aminotransferase) and ALT(alanine aminotransferase) in *Dock2$^{-/-}$* mice were measured at 72h after LPS injection. For all panels, error bars show the means ± SEM. ns: no significant, *$p < 0.05$, by an unpaired student t test. Data are representative of three independent experiments.

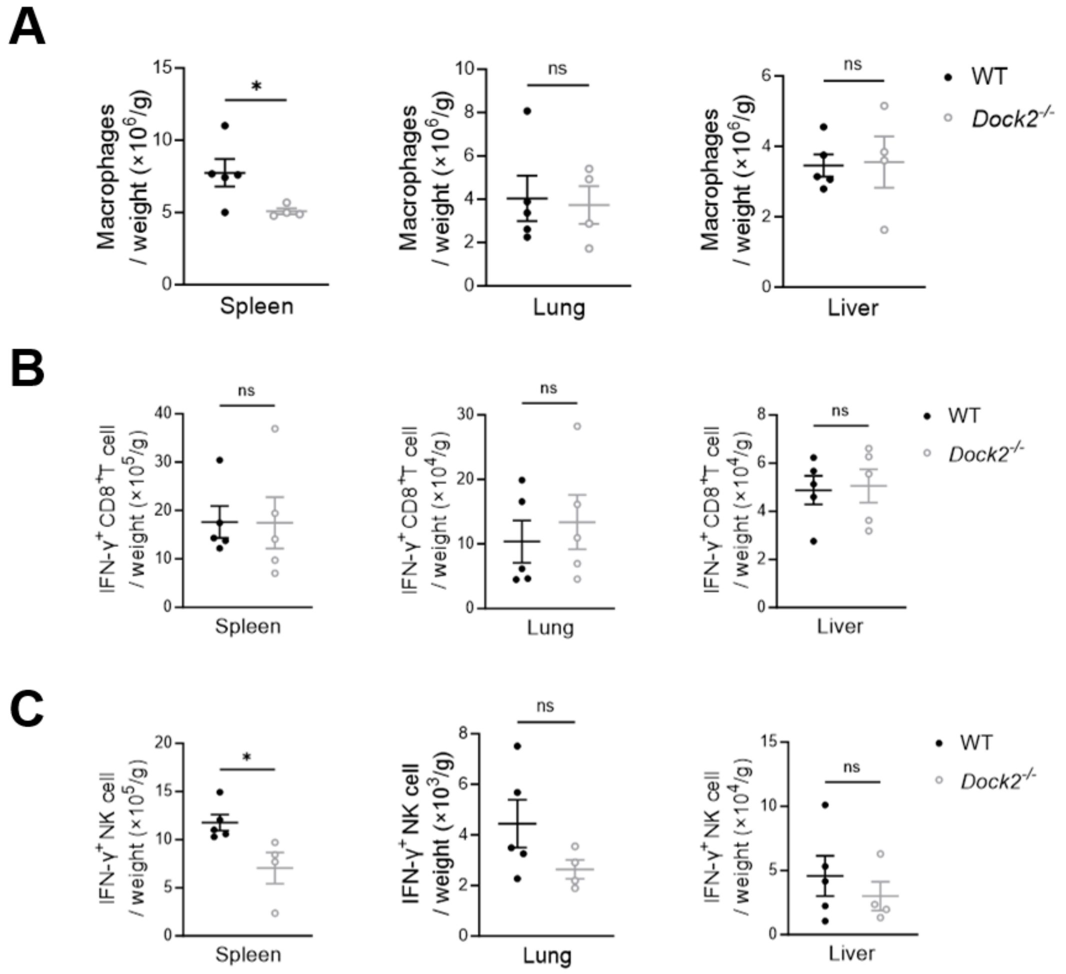

Supplemental Figure 2. Macrophages, IFN-γ^{+} CD8^{+}T cells and IFN-γ^{+} NK cells in *Dock2$^{-/-}$* mice under LPS induced sepsis. *Dock2$^{-/-}$* mice and WT littermates (n ⩾ 4/group) i.p. injected with 5 mg/kg of LPS as in Fig.1C. On day 3 after injection, mice were anesthetized and spleen, lung and liver were harvested and immune cell isolation for flow cytometric analysis. Relative numbers of Macrophages (Live&Dead^{-}CD11b^{+} F4/80^{+}) (A), IFN-γ^{+} NK cells (Live&Dead^{-}CD3^{-}NK1.1^{+}IFN-γ^{+}) (B) and IFN-γ^{+} CD8^{+}T cells (Live&Dead^{-}CD3^{+}CD8^{+}IFN-γ^{+}) (C) between WT and *Dock2$^{-/-}$* mice. For all panels, error bars show the means ± SEM. ns: no significant, *$p < 0.05$ by an unpaired student t test. Data are representative of three independent experiments.

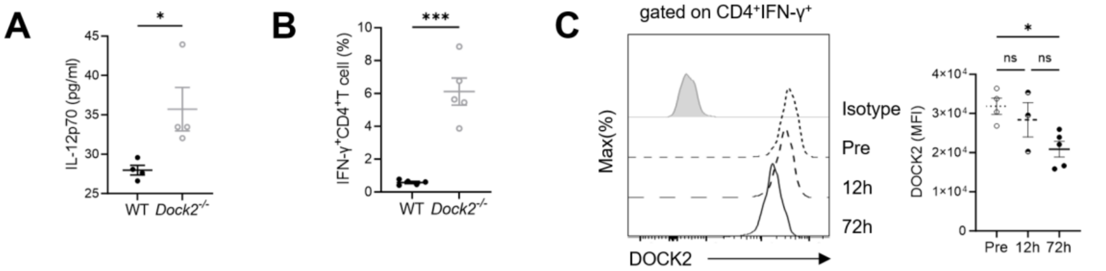

Supplemental Figure 4. DOCK2 has an important role in the host immune response to septic inflammation. *Dock2*$^{-/-}$ mice and WT littermates (n ⩾ 4/group) i.p. injected with 5 mg/kg of LPS as in Fig.1C. On 12 or 72 hours after injection, mice were anesthetized and spleen and serum were harvested and immune cell isolation for flow cytometric analysis. (A) The serum concentrations of IL-12p70 in *Dock2*$^{-/-}$ mice was measured at 12h after LPS injection. (B) Statistical analysis on the percentage of IFN-γ$^+$CD4$^+$ T cells (Live&Dead$^-$CD3$^+$CD4$^+$IFN-γ$^+$) in the spleen 12h after LPS injection are shown. (C) Flow cytometric analyses of DOCK2 protein expression in IFN-γ$^+$CD4$^+$ T cells of spleen. For all panels, error bars show the means ± SEM. ns: no significant, *$p < 0.05$, **$p < 0.01$ by an unpaired student t test. Data are representative of three independent experiments.

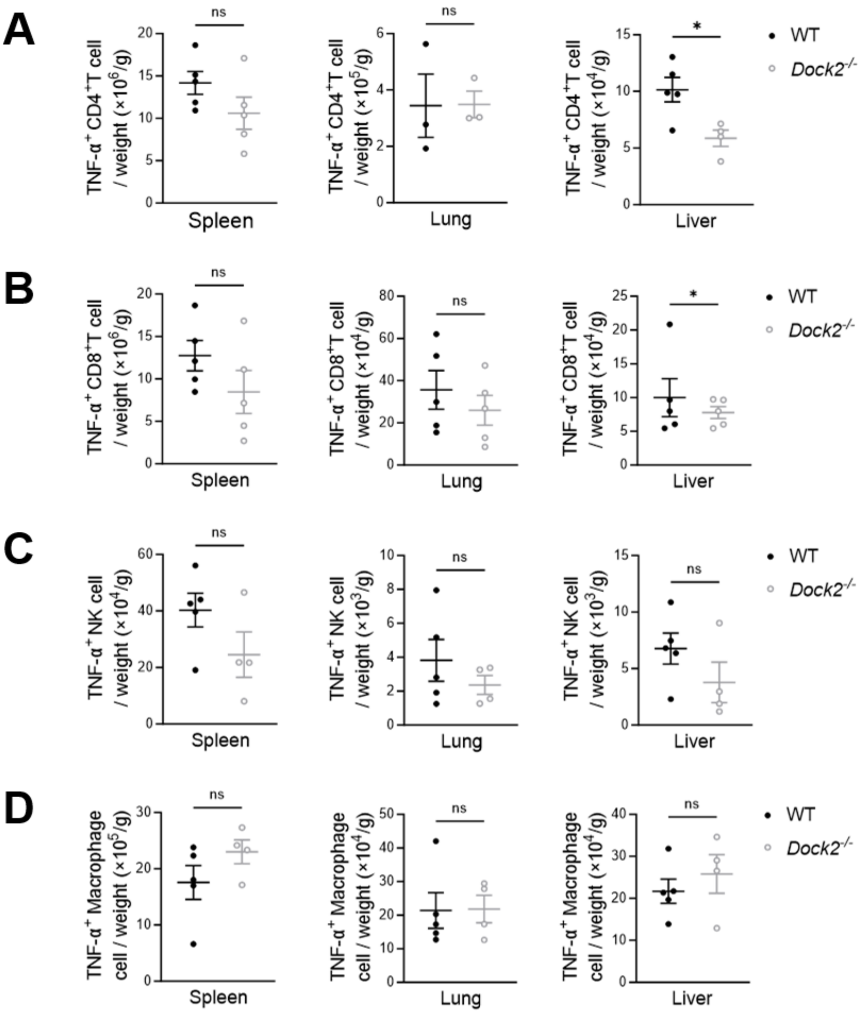

Supplemental Figure 5. TNF-α$^+$ CD4$^+$ T, CD8$^+$ T, NK cells and Macrophages in *Dock2*$^{-/-}$ mice under LPS induced sepsis. *Dock2*$^{-/-}$ mice and WT littermates (n ⩾ 4/group) i.p. injected with 5 mg/kg of LPS as in Fig.1C. On day 3 after injection, mice were anesthetized and peripheral blood, spleen, lung and liver were harvested and immune cell isolation for flow cytometric analysis. Relative numbers of TNF-α$^+$ CD4$^+$ T cells (Live&Dead$^-$CD3$^+$CD4$^+$TNF-α$^+$) (A), TNF-α$^+$ CD8$^+$ T cells (Live&Dead$^-$CD3$^+$ CD8$^+$TNF-α$^+$) (B), TNF-α$^+$ NK cells (Live&Dead$^-$CD3$^-$NK1.1$^+$TNF-α$^+$) (C) and TNF-α$^+$ Macrophages (Live&Dead$^-$CD11b$^+$F4/80$^+$TNF-α$^+$) (D) between WT and *Dock2*$^{-/-}$ mice. For all panels, error bars show the means ± SEM. ns: no significant, *$p < 0.05$ by an unpaired student t test. Data are representative of three independent experiments.

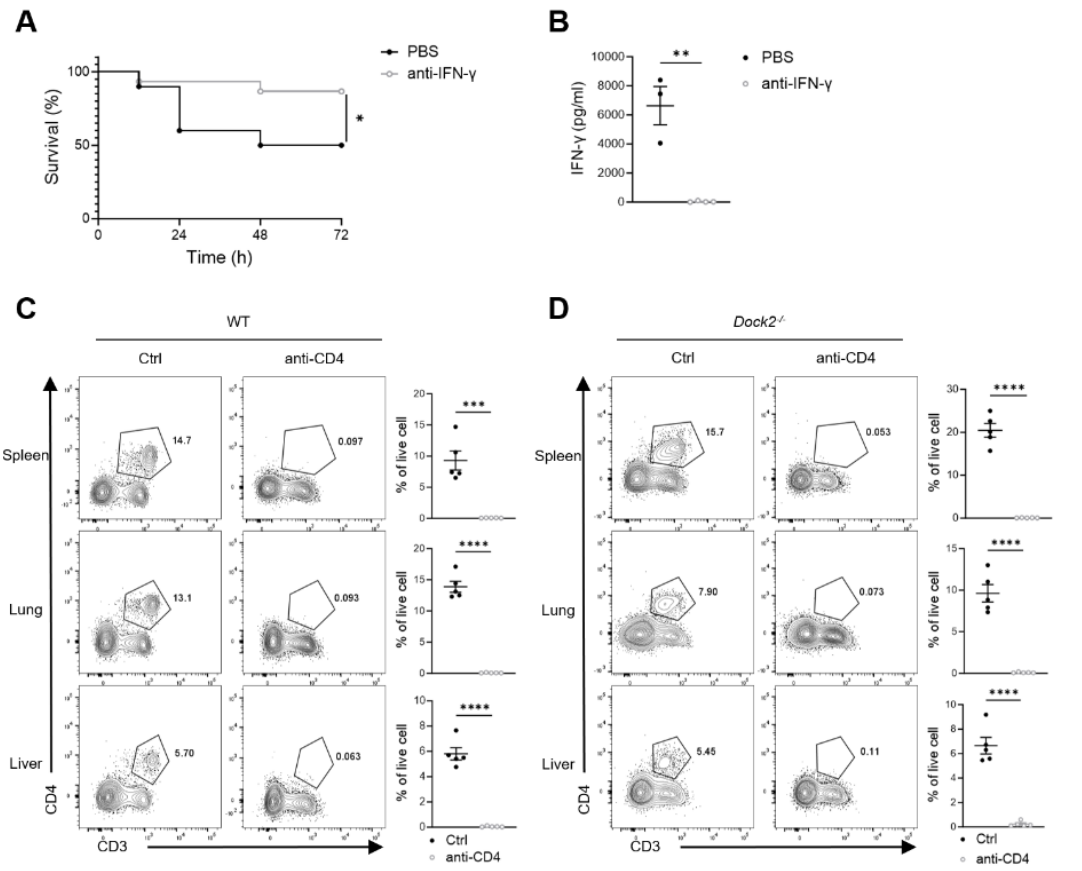

Supplemental Figure 6. Validation of the immune cell depletion efficiency. (A-B) *Dock2*$^{-/-}$ mice i.p. injected with LPS (5 mg/kg of body weight) and then treatment with PBS (n = 10) or anti-IFN-γ antibody (300 μg/mice) (n = 15) 4h later. Survival of mice was monitored for 72h. (A) The survival rate of LPS-induced sepsis mice expressed as a percentage. (B) The serum concentrations of IFN-γ were measured at 12h after LPS injection. Flow cytometry analysis of changes in immune cells from spleen, lung and liver of WT (C) and *Dock2*$^{-/-}$ mice (D) underwent CD4^{+} T cells depletion as indicated (n = 5/group). For all panels, error bars show the means ± SEM. *$p < 0.05$, **$p < 0.01$, ***$p < 0.001$, ****$p < 0.0001$ by an unpaired student t test. Data are representative of three independent experiments.
